# Remembering Dr Mark/Meir Dvorjetski: Physician, Survivor, Teacher, Historian, and Pioneer of Shoah Medicine Research

**DOI:** 10.5041/RMMJ.10505

**Published:** 2023-07-31

**Authors:** Deborah E.-S. Hemstreet, George M. Weisz

**Affiliations:** 1 English Communications Coordinator, Rambam Health Care Campus, Haifa, Israel; 2 Editorial Assistant, Rambam Maimonides Medical Journal, Haifa, Israel; 3 School of Humanities, University of New South Wales, Sydney, Australia; 4 School of Humanities, University of New England, Armidale, Australia

**Keywords:** biological destruction, Dvorjetski, Holocaust medicine, pathology of deportation, recovery

## Abstract

Meir Dvorjetski was a Holocaust survivor, teacher, and historian. He is best remembered for his descriptions of the medicine practiced by the Nazis during World War II, as well as the diseases, disorders, syndromes, and deaths resulting from such practice—particularly, though not solely, on the Jewish race. Dvorjetski’s contributions to Holocaust research at Bar-Ilan University in Israel, his underground partisan work, his contributions to society, and his testimony at the Eichmann trial have all been well documented. However, his earlier years—including his survival of the Holocaust, and his less-known medical achievements and contributions to historical records regarding the Holocaust—have not been covered as thoroughly. These latter items are the focus of this paper, with a closing commentary on the relevance of his work for the 21st century.

## INTRODUCTION

**Figure 1:**
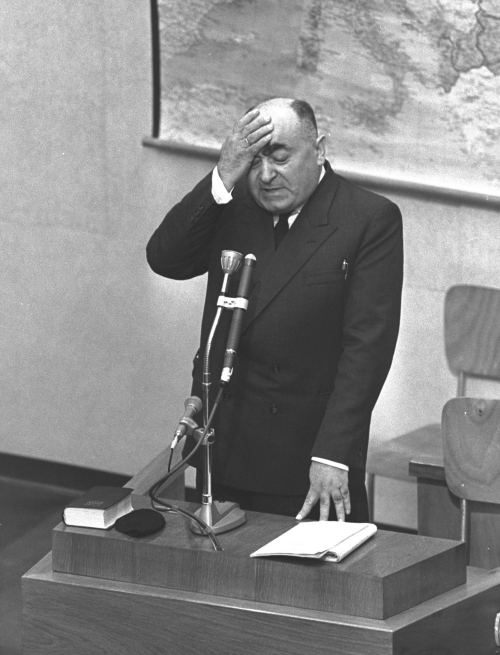
Photo of Dr Dvorjetski, a Witness for the Prosecution, During His Testimony at the Trial of Nazi War Criminal Adolf Eichmann, Held at Beit Ha’Am in Jerusalem. Photo courtesy of the Government Press Office, Israel.

Dr Meir (Mark) Dvorjetski (Figure 1), an intriguing man with a commanding presence, was the pioneer of Holocaust medicine research. Against all odds, he survived internments in ten different camps under the Nazi regime. As a survivor Dvorjetski considered it his obligation “to tell all that he saw in the bottom of hell.”^1^^(p12)^,^2^^(p50)^ Indeed, he undertook documentation of everything he observed, heard, and learned regarding that regime’s implementation of the final solution on its victims—both those who were killed and those who survived.

Dvorjetski wrote in multiple languages, including English, French, Hebrew, and Yiddish. Many of his writings were published in journals and newspapers no longer in print nor easily accessible to the public. Hence, many of his observations are inaccessible to today’s broader international medical community.^3^ His detailed accounts range from the medical conditions encountered in the ghettos and concentration camps, particularly with regard to starvation and post-liberation recovery and adjustment, to the subsequent development of early and late organic and psychological syndromes.^4^–^6^ Although today’s understanding regarding some of these topics has changed, his writings remain relevant for modern medical practice, particularly with regard to the effects on victims of starvation and/or lengthy unmerited incarceration or deportation. Many of his observations, most of which were not translated into English, can be extended far beyond Holocaust survivors and their medical care, to all victims of the horrific genocides of modernity and their subsequent caregivers. Just as important, Dvorjetski’s writings serve as a stark warning of the moral dilemmas faced by medical professionals living under totalitarian regimes.

Dvorjetski’s life has been examined by many historians.^3^,^4^ He is best known for his underground partisan work, his contributions to society, his testimonies at the Eichmann trial which were donated to the Yad Vashem archives, and his contributions to Holocaust research as an instructor at Israel’s Bar-Ilan University, where he headed the School of History of European Jewry and Holocaust Research. However, much less has been written about his earlier years, his experiences during the Holocaust, and his subsequent medical achievements through his contributions to the historical records regarding Holocaust medicine. This paper seeks to provide a brief overview of these items, and fill that gap.

## EARLY LIFE

Meir Dvorjetski, originally named Mark Dvorzecki, was born in Vilna, Poland, in 1908. He and his siblings, Lisa, Sima, and Shifra, were raised in a Yiddish-speaking home. Their father, Dov Ber Dvorzecki, was both a pharmacist and an ordained rabbi. Hence, all grew up in a religious environment with a Hebrew education.

After excelling in his studies at the Vilna Hebrew Gymnasium, Dvorjetski graduated from the Faculty of Sciences in the Nancy University in France at the age of 19, having focused on physics, chemistry, and the natural sciences.^5^ He went on to medical school at Vilna University, graduating in 1935, and began practicing medicine in Vilna.

Dvorjetski’s election to the Vilna City Council in 1939 testifies to his strong character and the respect he commanded at an early age. Dvorjetski continued to work as a physician during the first two years of World War II in Vilna, a hub for refugees, and intermittently under Soviet occupation; living conditions were bearable.^6^ However, shortly after the German invasion of the Soviet Union in June of 1941, mass murder of the Jews of Vilna began. By the end of August, 1941, the Jews were incarcerated in the Vilna ghetto.^7^

## SURVIVING THE HOLOCAUST

In September 1939, at the beginning of WWII, Dvorjetski was drafted as a medical officer by the Polish army, subsequently taken prisoner, and sent to a prisoner of war camp in Krakow.^1^ He managed to escape and returned to Vilna where he remained between September 1941 and September 1943.^2^

Dvorjetski proceeded to work in the Jewish Hospital, which was within the Vilna ghetto. There, he worked in internal medicine and children’s clinics and also headed the Epidemiology/Sanitation Department.

Ghetto life became increasingly difficult. Dvorjetski’s father, Dov Ber, died in the ghetto. When the ghetto was liquidated in September 1943, Dvorjetski’s mother, Zvia, also died. He was forced to observe the removal of the so-called “unfit” and “subhumans” to the Ponary murder pits. Those persons considered fit, such as Dvorjetski and other physicians, were deported to forced labor in Estonia. Although Dvorjetski’s wife, Miriam, and his sister volunteered to join him in Estonia, they both perished on the journey.

### Estonia

**Figure 2:**
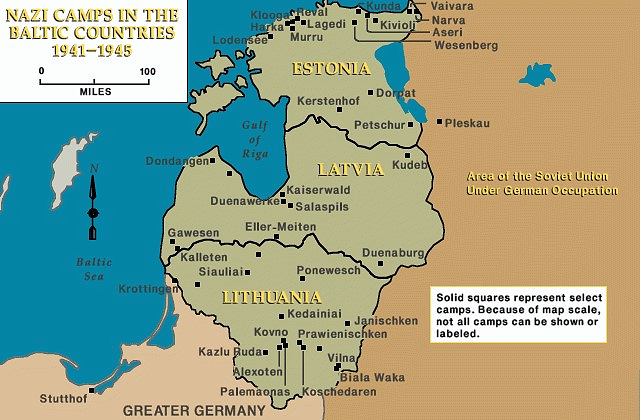
Locations of Large Nazi Camps in Estonia, Latvia, and Lithuania. Image courtesy of the United States Holocaust Memorial Museum.^8^

Twenty-two German camps were set up in this small country, as the occupied mines (metal, coal) required workers to carry out heavy physical labor (Figure 2).^9^ Dvorjetski was placed in the Vaivara camp; there were 70 people in each room, water was brought for showers once a week, and drinking water was obtained from boiled snow. Being a physician, he was forced to work triple duty—in the quarry breaking stone into gravel, cleaning the camp, and working as a physician. He would be transferred to several camps in the region, including Vivikoni, Vaivara, Johvi, Kureme, Godfiles, and finally Laguedi. In each location, Dvorjetski’s work ranged from digging trenches and bunkers to working in a lumber mill. But always his skills as a physician were required in some way.

Once, when performing medical duties at Vaivara, he hid a typhus diagnosis from the authorities. Dvorjetski was beaten by a *Schutzstaffel* (SS) officer and suffered fractured ribs and pleural effusion. Dvorjetski never forgot the SS officer who beat him: Dr. med. Franz von Bodmann, from a “noble” family. Bodmann held the paramilitary SS rank *Obersturmführer* and was the chief physician of Vaivara. When arrested after the war, Bodmann committed suicide.^10^

Like other prisoners, Dvorjetski experienced beatings and some of the diseases rampant in the camps, such as dysentery.

### Transfer to Germany

**Figure 3:**
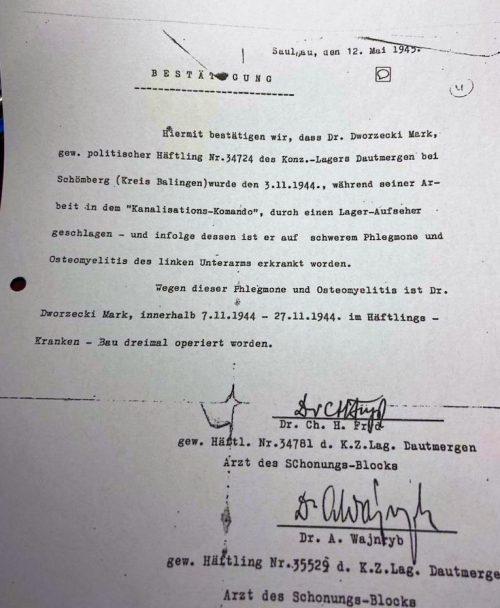
Hospital Discharge Card Following Medical Treatment in the Dautmergen Concentration Camp. Unofficial Translation: Saulsau, 12 May 1945. Confirmation. We confirm here that Dr Dworzecki Mark, i.e. political prisoner no. 34724 from the concentration camp Dautmergen at Schomberg (Kreis Balingen) was beaten on the 3.11.1944, during his work at the “Sewerage-Commando,” by a camp overseer—and as a result he became ill with severe phlegmon and osteomyelitis of the left lower arm. Because of phlegmon and osteomyelitis Dr Dworzecki Mark was operated three times during the time of 7.11.1944–27.11.1944 at the prisoners’ hospital. Signed by Dr. Ch. H. Fryd, i.e. Prisoner no. 34781 of the Concentration Camp Dautmergen, Physician of Convalescence Block; and Dr. A. Wajnryb, i.e. Prisoner no. 35529 of the Concentration Camp Dautmergen, Physician of Convalescence Block. Photo from the Archives of Yad Vashem.^11^

As geopolitical conditions changed and the Red Army came closer to Estonia, prisoners were transferred to the North Sea area toward Germany, namely to the Stutthof concentration camp; this one included gas chambers. Dvorjetski was assigned to carting coal loaded on stretchers. In the autumn of 1944, he was placed on a freight train and underwent a 7-day transfer to southwest Germany and the Dautmergen subcamp of the notorious Natzweiler-Struthof. There, hard labor consisted of building canals. In November 1944, whilst protecting a friend, Dvorjetski was severely beaten, resulting in a fractured left forearm bone (ulna), complicated with sepsis, that required three surgical interventions (Figure 3). Fortunately, a secretly obtained antibacterial, sulphonamide (Prontosil), facilitated his healing.

Yet another camp transfer ensued. On April 23, 1945, as the prisoners were marching toward Dachau, Dvorjetski managed to escape into the forest and was subsequently freed by the French army.

### Dvorjetski’s Key to Survival

Dvorjetski’s documentation of his experiences comprises a list of dates and occurrences.^5^ However, Dvorjetski himself gives a clear indication of what it took to survive the horrors he experienced via acutely written observations of other survivors of the Holocaust. His descriptions reflect a clear understanding of the strength of character it took for survival—a strength of character that he clearly shared with other survivors. In his article, “Adjustment of detainees to camp and ghetto life,” Dvorjetski vividly describes the mentality and actions of those who sought to survive, escape, and who actively refused to submit to the inhumanity around them.^12^^(p206)^ Dvorjetski believed that the core to survival was that “Ethical values played a leading role in the adjustment of the prisoner to camp life, and gave him a far better chance of survival.”^12^^(p204)^

## LIBERATION AND LIFE’S WORK

Following liberation, Dvorjetski was severely undernourished, weighed 39 kg, had recovered from typhus exanthematous (Fleck fever), typhoid, erythematous dermatitis, gastrointestinal infection, and was hospitalized for a respiratory illness diagnosed as emphysema and asthma.^5^ After receiving medical care, Dvorjetski was discharged and headed for Paris.

In Paris, Dvorjetski learned that his family had not survived. Nevertheless, from the outset, his actions reflected a determination to direct his memories toward educating the world regarding what had happened and using his medical skills to benefit the survivors of the Holocaust. During the next 4 years, Dvorjetski established a new family with Chasia Gefen, a refugee who had been saved by hiding in the south of France. He became the editor of a Jewish publication, *Our Word*, and began to attract attention for his series of articles titled “Recurrent Thoughts.”^13^

Shoah scholar Konrad Kwiet believes that no exact date can be established for the first Shoah medicine-related publication, although numerous documents were collected after August of 1945 regarding the Shoah.^14^ The earliest book might be *Smoke Over Birkenau* by Seweryna Szmaglewska, which was published in August of 1945.^15^ Dvorjetski’s earliest known article is dated May 25, 1945. Written in Yiddish, it graphically describes the inner psychological world of a concentration camp prisoner just before being released.^16^ Dated only 17 days after the liberation, this may well be the first post-war publication related to the Shoah.

Dvorjetski narrowed the focus of his writing in subsequent months to what would soon be known as Shoah medicine. His first official publication, in French, was a sanitation report on the Vilna ghetto in 1946.^17^

Many of Dvorjetski’s original documents were not written in an international language nor published in academic journals; hence they remained, for the most part, read by few. However, his numerous other publications demonstrate a high-level yearly output, maintained steadily from 1948 until 1974.^3^^(pp478–87)^,^18^

The depth and breadth of Dvorjetski’s writings reveal a unique individual who had keenly observed and analyzed his experiences. This ranged from the Nazi policies and their implementation; the impact of those policies on their victims physically and mentally; observations regarding different survivor mentalities; and extension of this knowledge to the healthcare and well-being of the Holocaust survivors. Dvorjetski promoted Congresses on Shoah diseases and became actively involved in the rehabilitation and placement of refugees.

In November of 1949, Dvorjetski immigrated to Israel where he practiced general medicine for the rest of his life. In parallel, he became a social activist who fought for the refugees. In 1959, now going by the name of Meir Dvorjetski, he became the first head of Bar-Ilan University’s new School of History of European Jewry and Holocaust Research. His 172 collected publications in the Yad Vashem Archives include history, medical studies, conference presentations, books, and chapters in books. His book, *Yerushalayim de-Lita bi-meri ube-sho’ah* [Jerusalem of Lithuania, in revolt and in Shoah], led to Dvorjetski being awarded the Israel Prize for Social Sciences in 1953.^3^ He subsequently became particularly recognized worldwide for his testimony during the Eichmann trial in 1961.

Dvorjetski succumbed to heart disease in 1975. Chaim Lazar, who was a contemporary of Dvorjetski’s and shared with him a similar dedication to Holocaust research and commemoration, summarized Dvorjetski as being “a wonderful, colorful person, honest and straightforward, a very good friend and above all, a devoted husband and father.”^19^

## DVORJETSKI’S MOST IMPORTANT CONTRIBUTIONS TO MEDICINE

Much has been written about Dvorjetski’s contributions to Holocaust medicine regarding the so-called medicine practiced by the Nazis and the medical and psychological impact on the survivors. Dvorjetski provided in-depth analyses of the atrocities perpetrated upon the Nazis’ incarcerated victims and the long-term effects on the survivors. In addition to looking at the unique medical conditions that affected survivors, he examined critical psychological elements that were unique to Holocaust survivors, such as readjusting to a new society, feelings of homelessness, the impact of being a sole survivor in a given family, the psychological depression in those who could not believe they were really free, and the uncertainties of future survival such as whether or not there was a place to return to.^16^,^20^,^21^ He provided detailed accounts of ghetto living conditions and the resistance of the Jewish physicians both in circumventing rules and by creating their own means of sanitation, vaccinations, and disinfection to fight infectious diseases, improve health in general, and to save lives—particularly those of children.^9^,^20^,^21^

However, beyond all of the above, Dvorjetski made important contributions to concepts that remain relevant today, namely: the pathology of deportation, biological destruction, and planned starvation. These concepts are briefly discussed below.

### The Pathology of Deportation

The concept of a pathology of deportation was first developed by French doctors in the 1940s and related to the psychological and physical conditions found in survivors of extreme war-related conditions such as deportations and internment.^22^,^23^ Notably, this concept was first related to prisoners of war and not to Jewish concentration camp survivors.^24^^(p71)^ The evolution of the concept to medical and psychological applications relating to incarcerated Holocaust survivors is beyond the scope of this paper and can be read elsewhere.^24^

Of import is Dvorjetski’s contribution which expanded upon the meaning, depth, and breadth of the pathology of deportation, particularly as it related to Holocaust survivors.^25^,^26^ Firstly, Dvorjetski did not believe that applying the pathology of deportation only to survivors who had been deported to concentration camps went far enough. He expanded the concept by calling it a Pathology of the Holocaust and included all phenomena discovered in those who were deported and transported to ghettos, Nazi concentration camps, and non-Nazi labor camps, as well as those who hid in bunkers or among the partisans. He emphasized that the pathology did not end with the war and was persisting in the lives of survivors 10 years later, and would persist throughout their lives as a result of all they had suffered. This post-Holocaust suffering he referred to in Hebrew as the pathological “residue” of the Holocaust period.^25^

In his examination of the pathology of the Holocaust, Dvorjetski justified his terminology by pointing out that one goal of scientific research in this area was to determine all of the biological and pathological phenomena emanating from the Holocaust period. He stressed they were “phenomena that we hope will never happen again, and for that reason, we hope they belong only to this history of the period of terror in Europe.”^25^^(p3)^

Tragically, Dvorjetski’s hope has not been realized. Hence, the second purpose that he gave for researching the pathology of the Holocaust (i.e. deportation), is more important than ever. This related to the future of the survivors and finding out what diseases, in particular, they were susceptible to, and what medical and social means could be used to promote the healing and rehabilitation of survivors who would continue to suffer as a result of their suffering during the Holocaust.^25^

In his writings related to the pathology of deportation, Dvorjetski also pointed out that critical contributors to the emotional, spiritual, or physical suffering of the survivors were the consequences of the biological destruction specifically aimed at the Jewish people and the pathology of hunger that they suffered due to the starvation strategies of the Nazis.

### Biological Destruction

In the context of the Holocaust, the concept of biological destruction related to the genetic purification of the German Nation. The German National Socialists aimed to prevent an “unhealthy” genetic inheritance. Preventing the undesired genetic inheritance relied heavily on the complicity of medical professionals.^27^

Dvorjetski translated his personal and observed experience as a physician in the Vilna ghetto and as a prisoner in both labor and death camps into a scientific analysis of the implementation of biological destruction, specifically (though not confined to) the Jewish people.^28^–^32^ In his book, *Europe Without Children*, this concept was examined with a particular focus on eugenics implementation on children.^29^

From the perspective of the authors of this paper, eugenics, as practiced on children, can be viewed as a kind of double Faustian pact. From Dvorjetski’s perspective, eugenics had a positive and a negative side. The positive side involved the Aryanization of so-called “orphaned” children. Abducted from Jewish Polish parents, children with Aryan features, i.e. blond hair and blue or green eyes, aged 8–10, were re-educated and eventually adopted by German families, an arguably humanitarian move since the children were allowed to live.

Negative eugenics lay on the other side of this presumed satanic pact. Dvorjetski observed that the negative side affected both German and Jewish children with physical or mental anomalies. They were prevented from transmitting pathological genes to subsequent generations (i.e. cleansing of the Fatherland) and subjected to either sterilization or extermination.^28^,^29^ In this way Aryan purity would be protected from inheritable diseases and disabilities including limb deformities, kyphoscoliosis and other spinal malformations, blindness, deafness, mania and depression, schizophrenia, Huntington’s chorea, and other neurological diseases. All such children were to be examined, diagnosed, and therefore condemned by the assessing medical practitioners.^27^,^30^

Dvorjetski provided an unemotional and scientific approach to the German techniques of sterilization or castration. Prevention of genetic transfer to future generations was by surgical severance of the male seminal tract, ligation of the fallopian tubes, intrauterine injection of inflammatory chemicals, or by oral, parenteral, or local administration of an extract from the Brazilian *Caladium seguinum* plant, known for its sterilization properties.^28^,^30^,^32^

Sterilization was also performed on the so-called feebleminded, whereas children who were defined as being incurably sick following a medical examination were euthanized. Dvorjetski described the nationwide institutions, mainly children’s hospitals, where euthanasia was performed on thousands of children. Experimentation was introduced without consent on patients in various institutions as well as in the concentration camps. Physical exams were performed by medical officers including Mengele, Clauberg, Schumann, Pokorny, and Verschuer, to name a few. However, the techniques used for sterilization, and later on for euthanasia, proved to be too impractical and slow, and not totally effective. Therefore, new and more effective methodologies were developed, such as extermination by carbon monoxide gas chambers. Hence, a well-designed program for biological destruction was developed and implemented.^28^,^29^,^32^

The complicity of medical personnel in the program’s development led to Dvorjetski’s critique of the German Medical Association for their abrogation of ethics and performance of criminal acts for which they faced no legal consequences.^33^ It is important to keep in mind that Dvorjetski was cognizant of the history of euthanasia as practiced under the Fuehrer’s rule. The first such case was performed in the Leipzig Children’s Hospital in 1939. The father of a newborn with severe physical anomalies (blind, immobile, having only one limb, and no signs of mental capacity) had asked for the euthanization of what he called a monster, and its return for burial. The medical staff refused and the father sought Hitler’s permission to do so. The Fuehrer’s response was immediate. He sent his private physician, Karl Brandt, to handle the situation. Permission was granted, together with impunity from any legal action; the child was euthanized, and the body returned to the parents.^27^,^34^

Despite their oath to protect lives, medical professionals actively supported the eugenics program. Encouraged by opportunities offered by the Nazi party, some 34% of the medical community joined the party, 7% of whom would also join the SS.^27^

At the 1947 founding conference of the World Medical Association, Dvorjetski demanded to “Throw the Anathema Against Murderer-Doctors.”^33^ His motion did not pass—then; however, he proved to be a visionary in his demand. In 1992, the previous SS doctor, Hans Joachim Sewering, after a 20-year presidency of the German Medical Association, was elected president of the World Medical Association. The global outrage forced Sewering to withdraw; his signature ordering the transport of 900 children to euthanasia remained as an undeniably damning document. Nevertheless, it was only in 2012 that the German Medical Association unanimously acknowledged responsibility for the crimes—without mentioning in their declaration the ethnicity of any of their victims.^35^

### Planned Starvation

It is perhaps an understatement to note that planned starvation was not only a methodology used by the Nazis to implement biological destruction; it was, additionally, a major contributor to the pathology of deportation noted in Holocaust survivors.^36^ Raphael Lemkin, who first coined the term genocide, considered starvation to be one tool used by the Nazis for the mass murder of the Jewish people.^37^,^38^ Lemkin did not have to look far for proof. Already in 1943, Field Marshal Gerd von Rundstedt had declared that planned starvation was better than machine guns for eradication of civil life in enemy countries.^38^–^40^

The systematic implementation of planned starvation by the Nazis has been well documented.^39^–^42^ It was implemented on all who were considered to be enemies. However, a specific plan was developed for use on the Jews. With reference to the ghettoization of the Jews in occupied Poland, the Nazi Governor Hans Frank wrote in his diary: “That we sentence 1.2 million Jews to die of hunger should be noted only marginally.”^43^

However, Dvorjetski provides historical details with scientific accuracy. He documented the impact of planned starvation not only on morbidity and mortality, but also the observed psychological, mental, and spiritual effects on its victims.^41^,^44^ He referred to the most extremely affected victims as “Muselman,” a recognized term at the time, which referred to concentration camp prisoners suffering from the combined effects of starvation (hunger disease) and exhaustion. These individuals had become completely indifferent and apathetic, having lost all hope for rescue.^12^ Dvorjetski observed that the degree of starvation was progressive, ranging from subnutrition of incarcerated populations, such as in the Vilna ghetto, progressing to the starvation experienced in the concentration camps, which ended with the most extreme case of the Muselman.^21^,^41^,^44^

Dvorjetski provided a scientific analysis of nutritional requirements for males and females, as opposed to that actually provided, as well as the basic metabolic requirements for inactive males (1680 calories/day) and females (1440 calories/day). This caloric requirement increased by 100% just for walking; moderate physical activities required 200% more calories per day, and heavy physical labor demanded a 400% increase—just for survival.^41^,^44^ However, according to Dvorjetski, the nutrition actually provided in the ghettos was 1800 calories/day, and this was progressively reduced, such that death could be expected within months, or in case of heavy labor, within weeks. Using data obtained from the Ringelblum Archives, Dvorjetski calculated the actual versus provided nutrition in the ghettos (Table 1).^31^ The same archives also provided data regarding the much lower caloric allotments in the concentration camps, which enabled Dvorjetski to calculate the daily caloric supply (Table 2).

**Table 1:** Actual versus Provided Nutrition in the Ghettos as Calculated by Dvorjetski.^31^

Nutritional Component	Standard Dietary Requirements	% Provided as of January 1941	% Provided by August 1941
Albumin	55 g/day	7%	5.3%
Fat	42 g/day	11%	9.5%
Carbohydrates	430 g/day	7%	5.4%

**Table 2:** Daily Caloric Allotments in the Main Concentration Camps.^31^

Concentration Camp	Calories per Day
Auschwitz I	1,100
Auschwitz II	900
Auschwitz III	1,000
Buchenwald	1,050
Dachau	535
Stutthof	1,300
Maidanek	1,000

The effects of starvation on children were particularly painful to Dvorjetski. He observed the children becoming joyless and apathetic, with slowed movement and retarded growth development, lying on their sides in the streets, legs folded into a fetal position. The children were hypothermic and had lost 50% of their weight. Many presented with enlarged lymph nodes, abdominal edema, and lower extremities with muscular atrophy. The children failed to enter healthy puberty (no menses, testicular atrophy). Broken bones did not heal, and many suffered from rickets or osteopenia, bradycardia, hypotension, and more. Ultimately—they died.^44^

Their suffering presented a moral dilemma to the ghetto physicians. In a more graphic essay written in Yiddish, Dvorjetski wrote that 75% of the children suffered from “struma” (enlarged thyroid) and recalls: “I still hear the heated discussion—how many drops [of iodine] should one give to the children, when in a few days or so their murder awaits them.”^3^^(p447)^

Dvorjetski also discussed the long-term results of planned starvation, which extended well past the liberation.^45^,^46^ Within a few months of May 8, 1945 (liberation), some 30,000 survivors died. Dvorjetski was hard-pressed to explain their course of rehabilitation. He noted that the fatalities were due to depressed cardiac, pulmonary, or immunological systems—ultimately the result of prolonged deprivation. The subsequent scientific explanation of refeeding syndrome, which included electrolyte imbalances as well as respiratory and cardiac failure, arrhythmias, seizures, coma, and eventual death, would be found and understood too late to be of help.^47^

## CONCLUSION

Ultimately, Dvorjetski’s research provided valuable information regarding the psychological and medical rehabilitation and treatment of Holocaust survivors. His in-depth observations of the effects of the trauma of deportation and the Nazi programs for biological destruction and planned starvation informed the world of horrors experienced that could not be ignored. Perhaps, more importantly, Dvorjetski’s observations provided invaluable input for understanding the medical and psychological sequelae that would be noted in Holocaust survivors for decades to come.

Dvorjetski differed from many of his contemporaries in that he was able to translate his personal experience during the Holocaust into a communal one. By doing so, he accurately testified to the atrocities he witnessed, applied his knowledge to medical practice for the benefit of survivors, and provided information that remains relevant today for treating the mental distress and physical diseases resulting from inhumane treatment and incarceration. Sadly, such suffering did not cease with the end of World War II.

According to the United Nations High Commissioner for Refugees, as of 2022, 108.4 million people have been forcibly displaced worldwide.^48^ An in-depth understanding of the medical and psychological impact of the pathology of displacement is needed now, more than ever. It is beyond the scope of this paper to examine known incidents of genocide (which includes the concept of biological destruction) and of famine—many modern instances of which can be arguably considered as planned starvation. However, medical professionals should be challenged to study Meir (Mark) Dvorjetski’s research and writings with the aim of gaining a better understanding of the long-term effects of these experiences on themselves, their patients, and ultimately society.

## Abbreviations

SS: Schutzstaffel
WWII: World War 2
